# Preparation and Photophysical Characterization of N‑Substituted 7‑Nitro-2,1,3-benzoxadiazol-4-amine Derivatives

**DOI:** 10.1021/acsomega.5c07309

**Published:** 2025-10-13

**Authors:** Jeremy P. Bard, Audrey K. MacNair, Tiyaba J. Jamil, Hayley E. Covington

**Affiliations:** Department of Chemistry, 2400 Washington College, Chestertown, Maryland 21620, United States

## Abstract

The 7-nitro-2,1,3-benzoxadiazole (NBD) scaffold is a widely studied and well-respected fluorophore. It is used in many sensing and imaging applications, both as an independent molecule and in larger molecular systems as a fluorescent building block. The broad applicability of NBD is due to its small size and respectable photophysical properties. To integrate this scaffold into these larger systems, NBD is often converted into 4-amino NBD with a spacer group that then acts as a bridge to connect it to the rest of the molecule. While many examples of these systems exist, this story seeks to expand the library of 4-amino NBD derivatives available for future use in more complex molecular systems. This study highlights the synthesis of one new compound, a new synthesis of a previously reported compound, and the photophysical characterization of each in several solvents.

## Graphical abstract

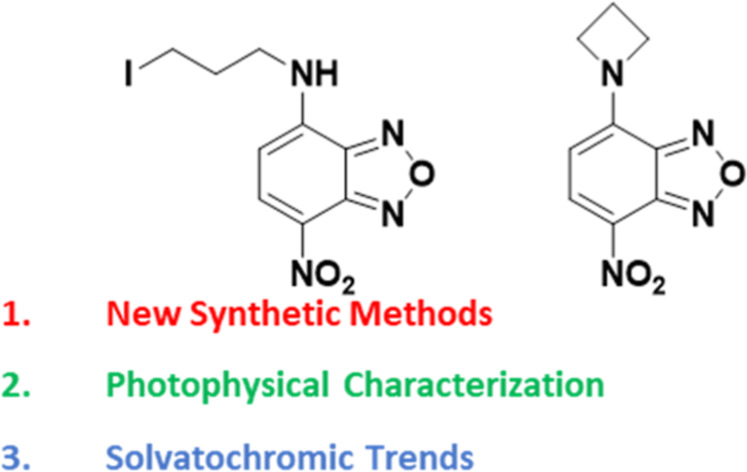

## Introduction

Due to their applications in medical, agricultural, and other imaging applications, small-molecule fluorophores have been a popular subject of research for many years.^1,3^ Because of the wide range of studies done so far, there exists an incredible variety of fluorophores that span the photophysical spectrum in their absorptions and emissions, exhibit unique physicochemical properties in the presence of different environments, and showcase niche applications in many areas. One fluorescent scaffold that has been studied and utilized a great deal is that of 7-nitro-2,1,3-benzoxadiazole (NBD) due to its small size, favorable photophysical properties, and environmental sensitivity (Figure 1a).^4,5^ Some of the simplest applications of the NBD scaffold for sensing purposes utilize the nonfluorescent 4-chloro-NBD as a probe for amines, amino acids, and thiols.^6,7^ NBD is often only a small building block within larger, more complex systems and is connected through various atoms in the 4-position including nitrogen, sulfur, and oxygen. All these atoms promote push–pull fluorescence when paired with the 7-nitro group, leading to enhanced quantum yields and red-shifted emissions. It has been integrated into structures that, among other things, detect analytes, exhibit fluorescence resonance energy transfer (FRET) properties, and label biological systems.^5,7–18^ The NBD moiety is often connected via alkyl, aryl, or heterocyclic linkers to the other components of the molecule. One key example showing this typical framework can be seen by a coumarin-NBD molecule that exhibits FRET characteristics and is utilized in H_2_S sensing (Figure 2b).^19^

**Figure 1:**
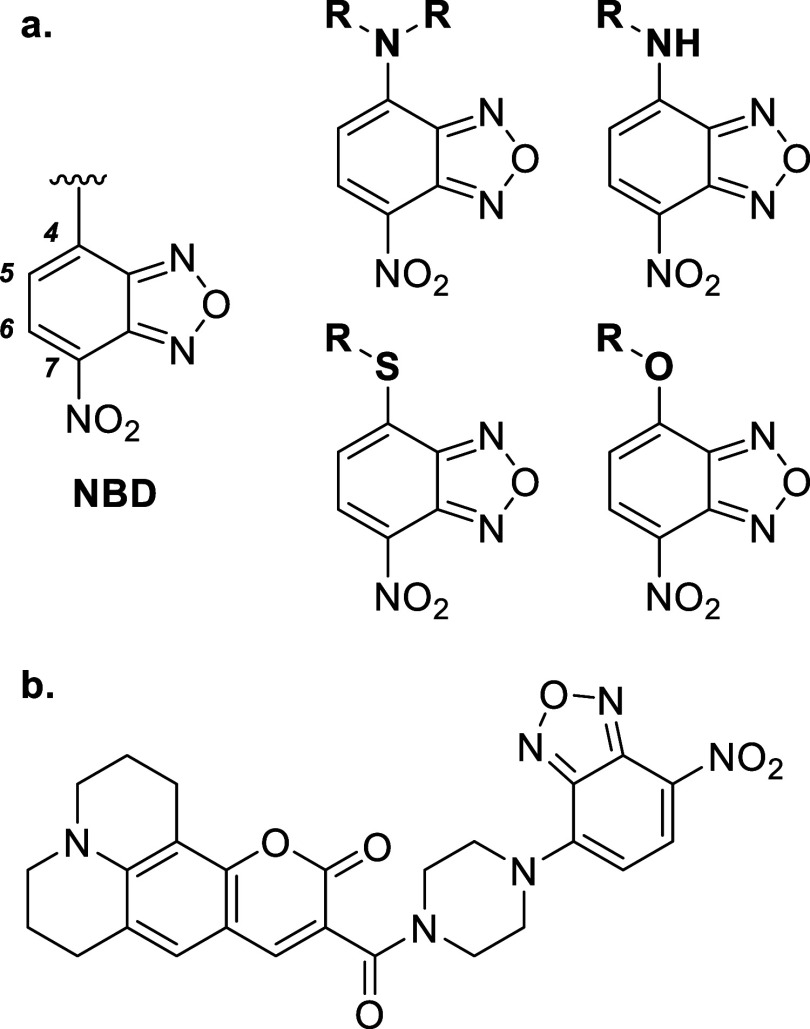
(a) Structure of NBD, with relevant carbon positions numbered alongside the examples of NBD derivatives and (b) representative NBD-containing molecule exhibiting how it is often covalently attached to other functional components in larger molecules through a short linker.

**Figure 2:**
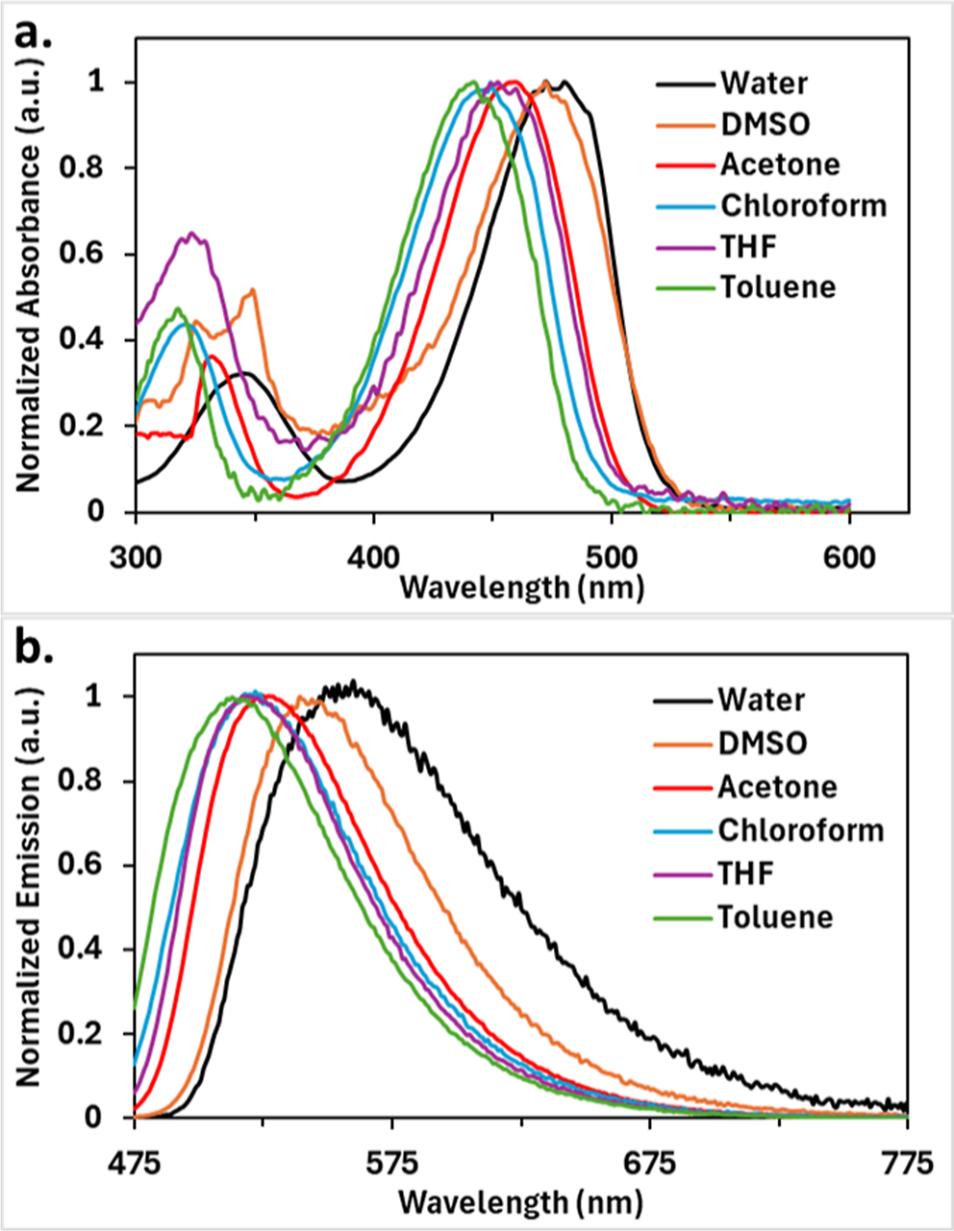
Stacked, normalized, (a) absorption and (b) emission spectra of **2** in various solvents at 298 K.

Due to the continued success of the NBD scaffold in these more sophisticated molecules, further development of functionalized NBD derivatives with different linkers and terminal functional groups holds value. By expanding the synthetic pathways for preparing both previously reported and new linker-functionalized NBD derivatives as well as determining the photophysical properties of these compounds in several environments, future studies in the field will have more options to build from when developing more complex NBD-containing structures. This work highlights the synthesis and photophysical characterization in various solvents of a new iodine-functionalized NBD derivative as well as new synthesis and characterization of a known azetidine-functionalized NBD derivative. The former shows promise as synthetic building blocks to facilitate future continued integration of the NBD skeleton into more complex molecular systems, as done previously with an analogous bromine-containing species,^20^ and the latter highlights an interesting and unexpected synthetic result.

## Results and Discussion

### Synthesis

#### Synthesis of *N*-(3-Iodopropyl)-7-nitro-2,1,3-benzoxadiazol-4-amine

The first goal of this study was to build upon a recent work that has shown the use of *N*-(3-bromopropyl)-7-nitro-2,1,3-benzoxadiazol-4-amine,^20^ **1**, as a reagent for the installation of NBD into larger systems.^21–25^ We sought to further amplify the reactivity of this molecule through the substitution of the bromine atom with an iodine atom, which would potentially allow it to react with a wider range of nucleophiles due to iodine’s increased leaving group ability. To accomplish this transformation, a Finkelstein reaction was performed by treating **1** with sodium iodide in refluxing acetone overnight (Scheme 1). After the mixture was concentrated in vacuo, resuspended in ethyl acetate, washed with water via liquid–liquid extraction, and subsequently concentrated in vacuo again, it afforded *N*-(3-iodopropyl)-7-nitro-2,1,3-benzoxadiazol-4-amine, **2**, as a deep red solid in an excellent 98% yield.

**Scheme 1:**
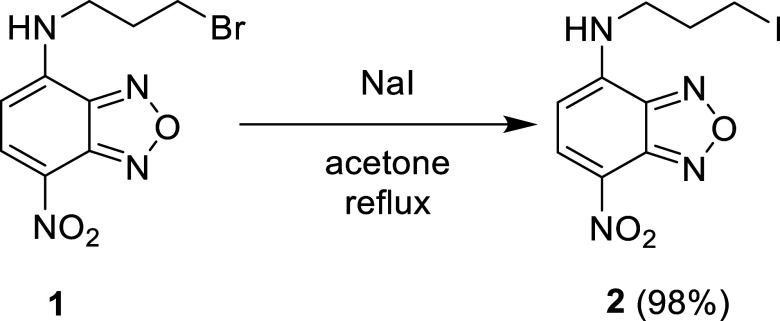
Synthesis of **2**

High-resolution mass spectrometry (HRMS) and nuclear magnetic resonance (NMR) studies confirm the structure of **2** (Figures S4 and S5). The HRMS-ESI­(+) analysis shows a large peak at 370.9614 *m*/*z*, which corresponds to the [M + Na]^+^ molecular ion peak that has an expected *m*/*z* value of 370.9612 for [C_9_H_9_IN_4_O_3_ + Na]^+^. The ^1^H NMR spectrum shows two doublets with areas of 1 at 8.51 and 6.28 ppm with coupling constants of 8.6 and 8.5 Hz, respectively, which correlate to the two NBD aromatic ring protons. The N–H signal shows up as a broad singlet of area 1 at 6.27 ppm, underneath one of the doublets mentioned above. The propyl chain is then illustrated by three peaks with areas of 2 each: a quartet with a coupling constant of 6.5 Hz at 3.69 ppm, a triplet with a coupling constant of 6.4 Hz at 3.31 ppm, and a quintet with a coupling constant of 6.6 Hz at 2.30 ppm. The ^13^C NMR spectrum shows two peaks at 136.4 and 99.1 ppm, corresponding to the NBD ring carbons containing hydrogen atoms. Four more aromatic peaks are observed at 144.5, 144.0, 143.6, and 124.8 ppm. Lastly, the three peaks correlating to the propyl chain are observed at 44.4, 31.6, and 1.6 ppm.

#### Synthesis of 4-(Azetidin-1-yl)-7-nitro-2,1,3-benzoxadiazole

The next goal of this study was to explore the other means of synthesizing the known compound *N*-(3-aminopropyl)-7-nitro-2,1,3-benzoxadiazol-4-amine. However, while attempting a substitution reaction between 7-nitro-2,1,3-benzoxadiazol-4-amine,^25^ **3**, and 3-bromopropylamine hydrobromide using triethylamine and 1,8-diazabicyclo[5.4.0]­undec-7-ene (DBU) in tetrahydrofuran (THF) and water overnight, 4-(azetidin-1-yl)-7-nitro-2,1,3-benzoxadiazole, **4**, was isolated instead as the main product via silica column chromatography (Scheme 2). Compound **4** was isolated as a crystalline, red solid in a modest 17% yield. While this compound has been reported previously,^27–30^ this reaction shows an interesting new method of preparing it that utilizes both a substitution and an intramolecular cyclization.

**Scheme 2:**
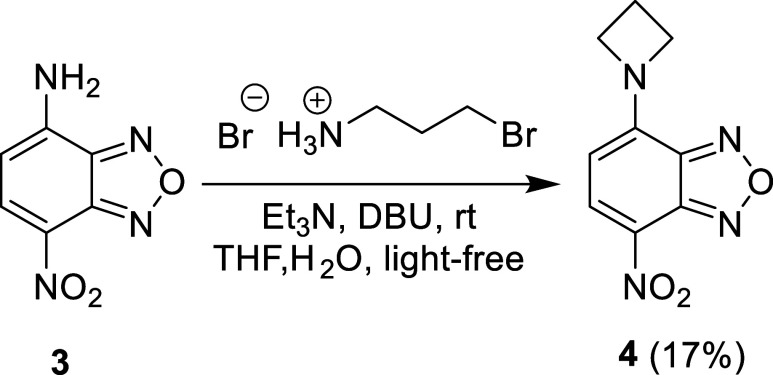
Synthesis of **4**

The structure of **4** was confirmed by HRMS and NMR studies (Figures S6 and S7). HRMS-ESI­(+) shows a large peak at 221.0667 *m*/*z*, which corresponds to the [M + H]^+^ molecular ion peak that has an expected *m*/*z* value of 221.0669 for [C_9_H_8_N_4_O_3_ + H]^+^. The ^1^H NMR spectrum shows a doublet of doublets at 8.47 ppm with an area of 1 and coupling constants of 8.9 and 3.4 Hz. There is a second doublet of doublets at 6.05 with an area of 1 and coupling constants of 9.0 and 3.4 Hz. These correlate with the two NBD ring C–H positions. There are two broad signals with areas of 2 at 4.77 and 4.42 ppm that correlate with the two methylene positions directly attached to azetidine. Lastly, a multiplet with an area of 2 is observed at 2.58 ppm for the remaining methylene group of the azetidine ring. The ^13^C NMR spectrum shows two peaks at 136.4 and 99.5 ppm, correlating to the two hydrogen-bearing carbons of the NBD ring. There are four more aromatic peaks at 145.2, 144.6, 143.7, and 119.1 ppm. The three peaks at 56.5, 53.0, and 16.5 ppm correlate to the three azetidine carbons.

### Photophysical Characterization

The absorption and emission properties of compounds **2** and **4** in solvents of varying polarities were then collected. The photophysical properties of starting material **1** were gathered for comparison purposes and are similar to those of **2** and **4** (Figure S1 and Table S1).

#### Photophysical Properties of **2**

In general, bathochromic shifts in absorption and emission values are observed (Figure 2 and Table 1). The maximum absorption values range from 443 nm in less polar toluene to 472 nm in more polar water. The maximum emission values range from 516 to 555 nm in toluene and water, respectively. The absorption coefficient values range from 15,000 to 27,000 M^–1^ cm^–1^. Quantum yields range from 95% down to 6%, indicating a significant drop when moving toward the more polar solvents, particularly water. This drop in quantum yield is expected, as the stronger dipoles of the more polar solvents better stabilize the fluorophore while it is in its excited state, thus leading to more nonradiative, or nonemissive, pathways to release the excess energy when returning to the ground state.^31^ Stokes shifts range from 66 to 83 nm, or 2700 to 3300 cm^–1^. Lastly, brightness values range from 1400 to 26,000 M^–1^ cm^–1^, due mainly to varying quantum yield values. When these findings were compared to those of **1**, slightly lower absorption coefficients and quantum yields were found for **1**.

**Table 1:** Photophysical Properties of **2** in Several Solvents^a^

solvent	λ_abs,max_ (nm)	ε_abs,max_ (M^–1^ cm^–1^)	λ_em_ (nm)	Stokes shift (nm/cm^–1^)	φ (%)	φ × ε (M^–1^ cm^–1^)
toluene	443	15,000	516	73/3200	93	14,000
THF	452	19,000	522	70/3000	91	17,000
chloroform	447	21,000	523	76/3300	89	19,000
acetone	458	27,000	524	66/2800	95	26,000
DMSO	471	22,000	539	68/2700	42	9200
water^b^	472	24,000	555	83/3200	6	1400

a All values collected at 298 K.

b Collected through the addition of concentrated DMSO stock solution of **2** into water, resulting in a <5% DMSO solution.

#### Photophysical Properties of **4**

Upon performing the same set of photophysical characterizations on **4**, trends similar to those of **2** were observed (Figure 3 and Table 2). The maximum absorption values range from 472 nm in toluene to 499 nm in water. The maximum emission values range from 532 to 553 nm in toluene and water, respectively. Absorption coefficient values are slightly lower in general than those for **2** and range from 13,000 to 24,000 M^–1^ cm^–1^. Quantum yields are also slightly reduced and range from 80% down to 4%. Stokes shifts values range from 60 to 85 nm, or 2200 to 2900 cm^–1^. These properties lead to reduced brightness values, ranging from 1000 to 11,000 M^–1^ cm^–1^, for **4** when compared to **1** and **2**.

**Figure 3:**
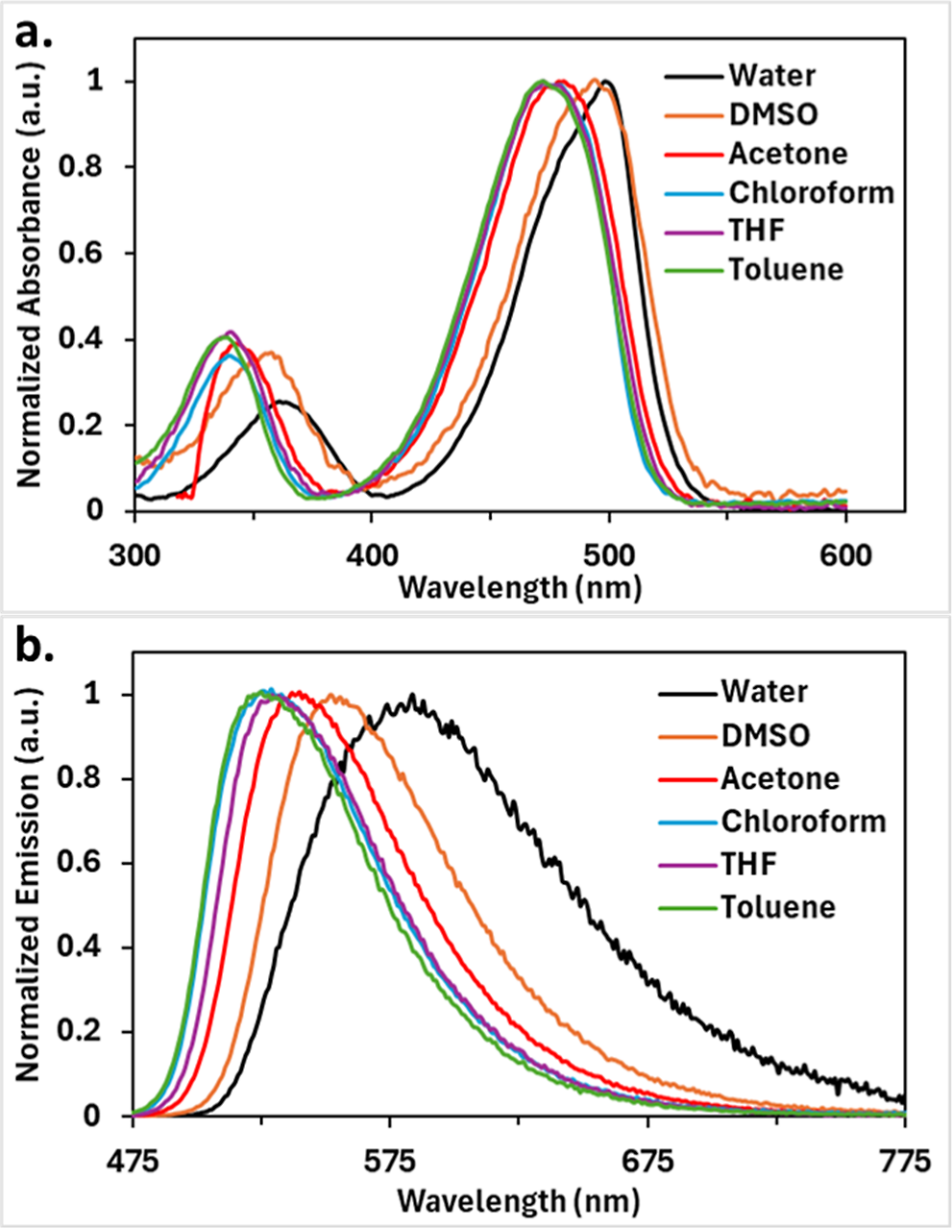
Stacked, normalized, (a) absorption and (b) emission spectra of **4** in various solvents at 298 K.

**Table 2:** Photophysical Properties of **4** in Several Solvents^a^

solvent	λ_abs,max_ (nm)	ε_abs,max_ (M^–1^ cm^–1^)	λ_em_ (nm)	Stokes shift (nm/cm^–1^)	φ (%)	φ × ε (M^–1^ cm^–1^)
toluene	472	13,000	532	60/2400	80	10,000
THF	473	13,000	533	60/2400	79	10,000
chloroform	472	15,000	535	63/2500	71	11,000
acetone	481	14,000	542	61/2300	53	7400
DMSO	493	17,000	553	60/2200	59	10,000
water^b^	499	24,000	584	85/2900	4	1000

a All values collected at 298 K.

b Collected through the addition of concentrated DMSO stock solution of **4** into water, resulting in a <5% DMSO solution.

#### Solvatochromic Trends in Photophysical Properties

When examining the absorption and emission spectra of **1**, **2**, and **4**, several trends arise. Primarily, all three fluorophores show consistent solvatochromic responses in both their absorption and emission maximum values. When plotting the absorption and emission maxima of **1**, **2**, and **4** against the *E*_*T*_(30) values of the solvents in which the data were collected,^32^ a positive correlation was seen in every instance (Figures S2, S3 and Table S2). Related to the impact of solvent polarity on the quantum yields above, solvent dipoles aligning with the excited states of fluorophores lower the energy of light absorbed and emitted, leading to the red-shifting in absorption and emission values, respectively.

Furthermore, the values for **4** are generally at wavelengths higher than those of **1** and **2**. Additionally, these plots highlight the striking similarities between the absorption and emission peaks found for compounds **1** and **2**, which are reasonable, as their structures are very similar to one another, with the identity of the halogen being the only difference.

## Experimental Section

NBD-Cl, sodium iodide, 3-bromopropylamine hydrobromide, triethylamine, DBU, silica gel, and all solvents were purchased from commercial vendors. Compounds **1** ^20^ and **3** ^26^ were prepared as previously described.

A Bruker AvanceCore nuclear magnetic resonance spectrometer (400 MHz) was used to gather structural spectra via ^1^H NMR (400 MHz) and ^13^C NMR (100 MHz). An Edinburgh Instruments FS5 spectrofluorometer excitating at 470 nm was used to gather all photophysical data. Quantum yields (φ) were determined through a comparison of the absorption and emission intensities of the analyte to those of a fluorescein standard dissolved in 0.1 M NaOH.^33^ An Agilent 6530 Q-TOF mass spectrometer was used to gather all of the HRMS data.

### Synthesis of **2**

Compound **1** (1.32 g, 4.38 mmol, 1 equiv) and sodium iodide (3.29 g, 21.9 mmol, 5 equiv) were dissolved in acetone (40 mL). The mixture was then heated at reflux for 16 h before being cooled to room temperature and concentrated in vacuo. The mixture was then dissolved in ethyl acetate (20 mL), washed with DI water (15 mL) in a separatory funnel three times, dried over sodium sulfate, and concentrated in vacuo to afford **2** in an exceptional yield (1.494 g, 98%) as a red solid: Melting point: 128.7–129.9 °C; ^1^H NMR (400 MHz, CDCl_3_, ppm) δ: 8.51 (d, *J* = 8.6 Hz, 1H), 6.28 (d, *J* = 8.5 Hz, 1H), 6.27 (s, broad, 1H), 3.69 (q, *J* = 6.5 Hz, 2H), 3.31 (t, *J* = 6.4 Hz, 2H), 2.30 (quint, *J* = 6.6 Hz, 2H); ^13^C­{^1^H} NMR (100 MHz, CDCl_3_, ppm) δ: 144.5, 144.0, 143.6, 136.4, 124.8, 99.1, 44.4, 31.6, 1.6; HRMS-ESI­(+) [M + Na]^+^ calcd for C_9_H_9_IN_4_O_3_, 370.9612; found, 370.9614.

### Synthesis of **4**

Compound **3** (0.872 g, 4.84 mmol, 1 equiv) was dissolved in THF (72 mL) before DBU (3.00 mL, 21.8 mmol, 4.5 equiv) and triethylamine (54 mL) were added. The vessel was then covered in a foil to protect it from light. In a separate container, 3-bromopropylamine hydrobromide (2.12 g, 9.69 mmol, 2 equiv) was dissolved in THF (72 mL) and water (6 mL). The solution of 3-bromopropylamine hydrobromide was then slowly added to the solution of **3,** and then the mixture was stirred for 48 h. Upon near-complete conversion by TLC, the reaction was then reduced in vacuo before three rounds of ethyl acetate (15 mL each) was added to the reaction mixture and subsequently removed in vacuo to help remove residual triethylamine. The mixture was purified via column chromatography (1:1:1 hexanes/EtOAc/CH_2_Cl_2_, *R*_f_ = 0.25) to afford **4** in a modest yield (181 mg, 17%) as a bright red solid: Melting point: 211.6–213.7 °C; ^1^H NMR (400 MHz, DMSO-*d*_6_, ppm) δ: 8.47 (dd, *J* = 8.9, 3.4 Hz, 1H), 6.05 (dd, *J* = 9.0, 3.4 Hz, 1H), 4.77 (s, broad, 2H), 4.42 (s, broad, 2H), 2.58 (m, 2H); ^13^C­{^1^H} NMR (100 MHz, DMSO-*d*_6_, ppm) δ: 145.2, 144.6, 143.7, 136.4, 119.1, 99.5, 56.5, 53.0, 16.5; HRMS-ESI­(+) [M + H]^+^ calcd for C_9_H_8_N_4_O_3_, 221.0669; found, 221.0667.

## Conclusions

This study shows the synthesis of a previously unreported **2** and an interesting new route for accessing previously reported **4**. The structures of these two species were confirmed by ^1^H NMR, ^13^C NMR, and HRMS. Photophysical characterization of **2** and **4** in several solvents ranging from nonpolar to polar revealed that both molecules possess similar photophysical properties and typical solvatochromic trends. Each molecule showed absorption values within the range of 443–499 nm, absorption coefficients from 13,000 to 27,000 M^–1^ cm^–1^, emission values within the range of 516–584 nm, and Stokes shifts values between 60 and 85 nm, or between 2200 and 3300 cm^–1^. Quantum yields also varied widely, where **2** showed values from 95 to 6%, and **4** showed values from 80 to 4%. The markedly low values are in water, however, which again align with the expected trends in more polar solvents. The new reactions making these compounds and a solid understanding of their photophysical properties across a range of solvents now expand the options available for researchers looking to integrate the NBD moiety into future molecular systems.

## Supplementary Material



## Notes

^1^H and ^13^C NMR spectra of compounds **2** and **4**; photophysical properties and spectra of **1**; and solvatochromic trends of **1**, **2**, and **4** (PDF)

## Abbreviations

NBD: 7-nitro-2,1,3-benzoxadiazole
FRET: fluorescence resonance energy transfer
HRMS: high-resolution mass spectrometry
NMR: nuclear magnetic resonance
HRMS-ESI­(+): high-resolution mass spectrometry using electrospray ionization in positive mode
DBU: 1,8-diazabicyclo­[5.4.0]­undec-7-ene
THF: tetrahydrofuran
