# Supplementary material for: Preparation and Photophysical Characterization of N‑Substituted 7‑Nitro-2,1,3-benzoxadiazol-4-amine Derivatives
Source: ACS Omega. 2025 Oct 13;10(42):50308–13. doi: 10.1021/acsomega.5c07309 (PMC12573151; doi:10.1021/acsomega.5c07309)
Supplement: Supplementary file 1 [file ao5c07309_si_001.pdf]

Supporting information for:

Preparation and Photophysical Characterization of *N*-substituted 7-Nitro-2,1,3-benzoxadiazol-4-amine Derivatives

Jeremy P. Bard,[*,†] Audrey K. MacNair,[†] Tiyaba J. Jamil,[†] Hayley E. Covington[†]

[†]Department of Chemistry, Washington College, Chestertown, MD 21620, United States

[*] Corresponding Author: Jeremy P. Bard (jbard2@washcoll.edu)

Table of Contents **Page**

1. Photophysical Properties and Spectra of **1** S2
2. Solvatochromic Trends of Dyes S3
3. References S4
4. Copies of NMR Spectra of **2** and **4** S5

1. <u>Photophysical Properties and Spectra of **1**</u>

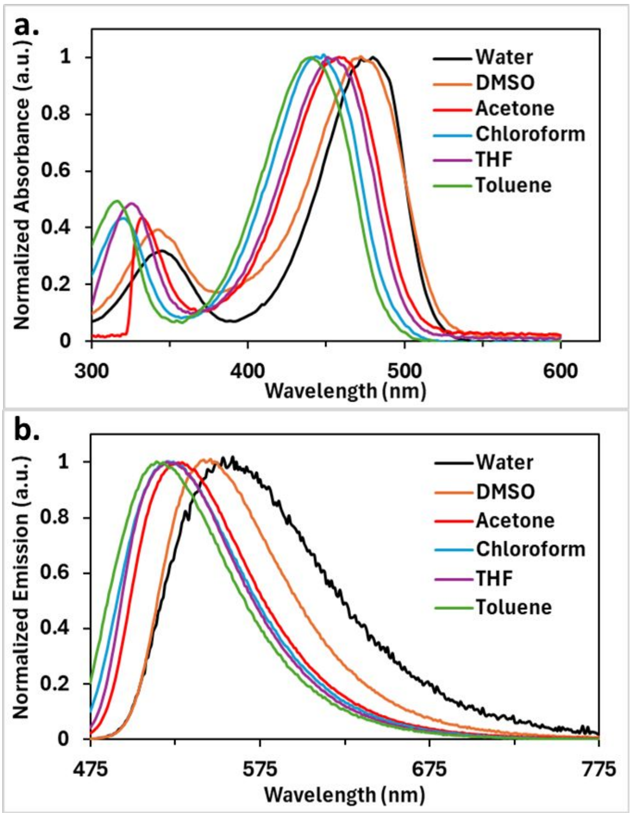

Figure S1. Stacked, normalized, (a.) absorption and (b.) emission spectra of **1** in various solvents at 298 K.

Table S1. Photophysical properties of **1**[1] in several solvents.[a]

Solvent	$\lambda_{abs,max}$ (nm)	$\varepsilon_{abs,max}$ ($M^{-1}\,cm^{-1}$)	λ_{em} (nm)	Stokes shift (nm/cm^{-1})	φ [c] (%)	$\varphi \times \varepsilon$ ($M^{-1}\,cm^{-1}$)
Toluene	440	9000	515	75/3300	75	6800
THF	451	11000	523	72/3100	72	7900
Chloroform	447	11000	522	75/3200	87	9600
Acetone	459	13000	527	68/2800	57	7400
DMSO	473	15000	546	73/2800	38	5700
Water[b]	480	16000	561	81/3000	7	1100

[a]All values collected at 298K. [b]Collected through the addition of concentrated DMSO stock solution of **1** into water, resulting in a <5% DMSO solution. [c]Determined through a comparison of the absorption and emission intensities of the analyte to those of a fluorescein standard dissolved in 0.1 M NaOH.[2]

2. <u>Solvatochromic Trends of Dyes</u>

Table S2. Compiled absorption and emission values and solvent $E_T(30)$ values.[a,b]

		**1**		**2**		**4**	
Solvent	$E_T(30)$ (kcal mol^{-1})	λ_{abs} (nm)	λ_{em} (nm)	λ_{abs} (nm)	λ_{em} (nm)	λ_{abs} (nm)	λ_{em} (nm)
Toluene	33.9	440	515	443	516	472	532
THF	37.4	451	523	452	522	473	533
Chloroform	39.1	447	522	447	523	472	535
Acetone	42.2	459	527	458	524	481	542
DMSO	45.1	473	546	471	539	493	553
Water[c]	63.1	480	561	472	555	499	584

[a]All values collected at 298K. [b]$E_T(30)$ values are previously reported.[3] [c]Collected through the addition of concentrated DMSO stock solution of the dye into water, resulting in a <5% DMSO solution.

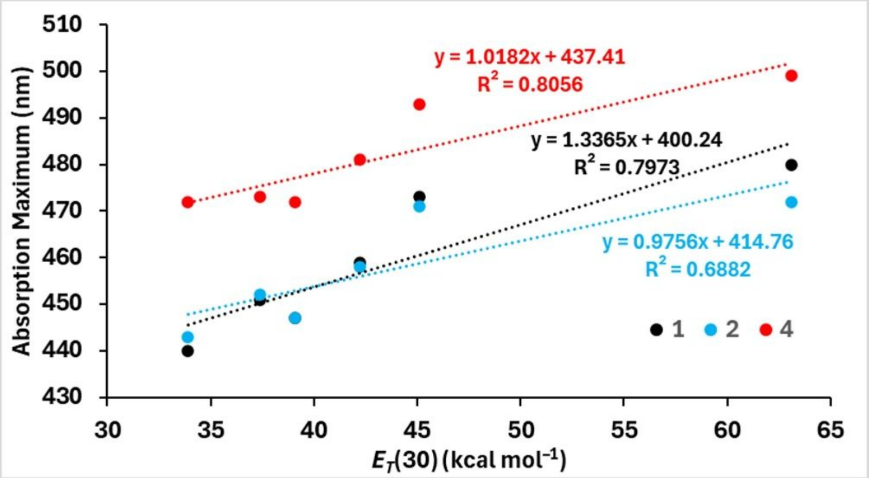

Figure S2. Absorption maximum vs solvent $E_T(30)$ trends for **1**, **2**, and **4**.

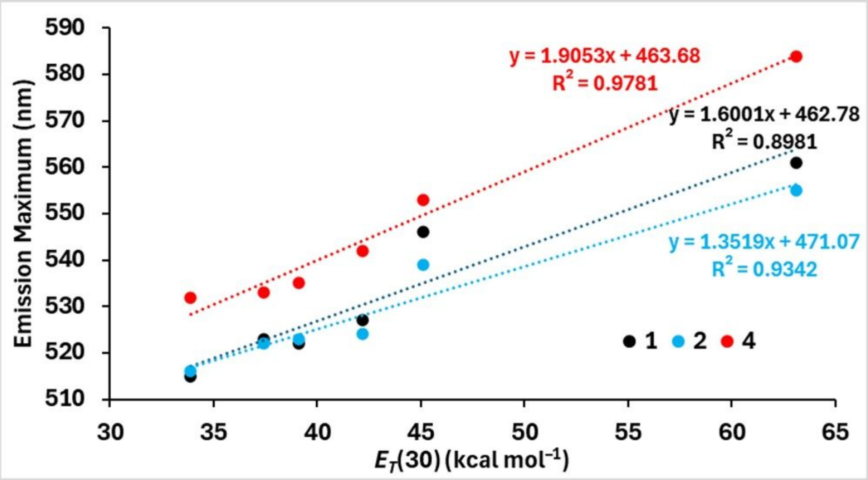

Figure S3. Emission maximum vs solvent $E_T(30)$ trends for **1**, **2**, and **4**.

3. <u>References</u>

1. de Munnik, M.; Lohans, C. T.; Langley, G. W.; Bon, C.; Brem, J.; Schofield, C. J. A Fluorescence-Based Assay for Screening β-Lactams Targeting the *Mycobacterium tuberculosis* Transpeptidase Ldt_{Mt2}. *Chembiochem* **2020**, *21*, 368–372

2. Brouwer, A. M. Standards for Photoluminescence Quantum Yield Measurements in Solution. *Pure Appl. Chem.* **2011**, *83*, 2213−2228

3. Reichardt, C. Solvatochromic Dyes as Solvent Polarity Indicators. *Chem. Rev.* **1994**, *94*, 2319−2358.

3. Copies of NMR Spectra of **2** and **4**

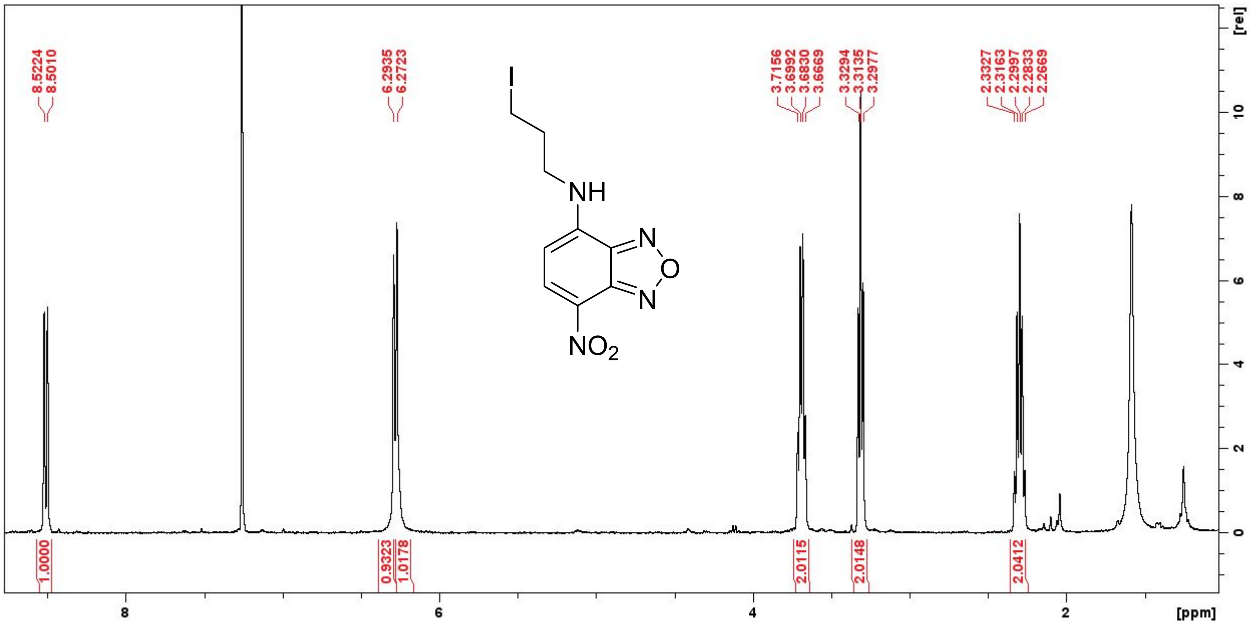

Figure S4. ^{1}H NMR spectrum of **2** in $CDCl_3$.

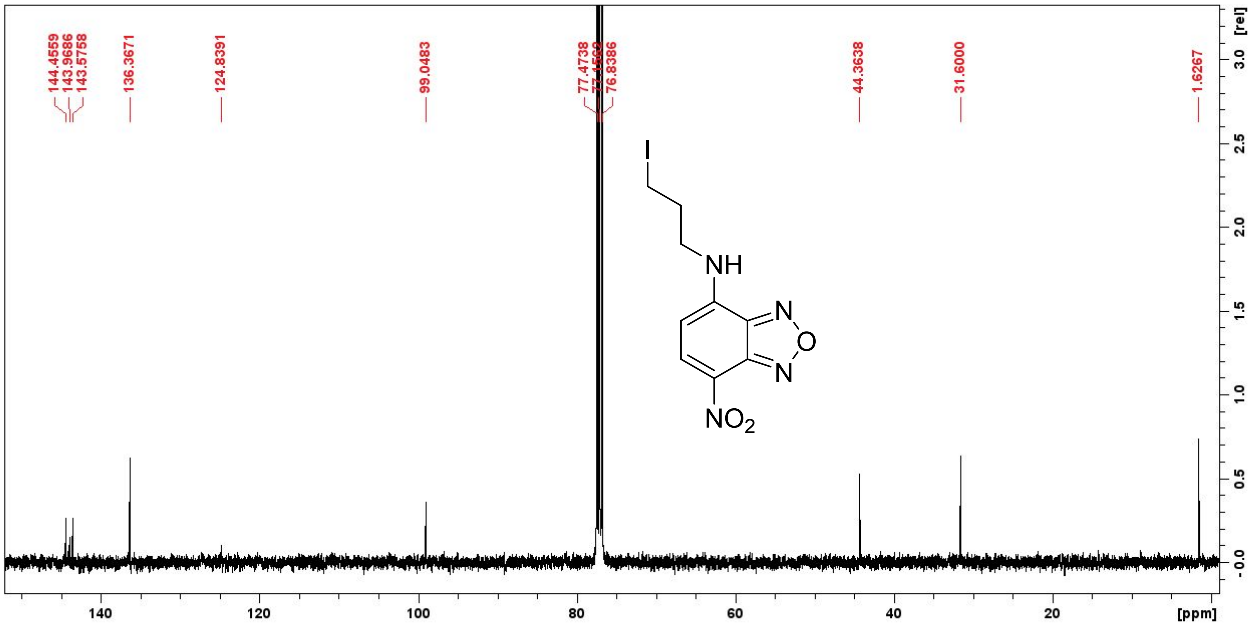

Figure S5. ^{13}C NMR spectrum of **2** in $CDCl_3$.

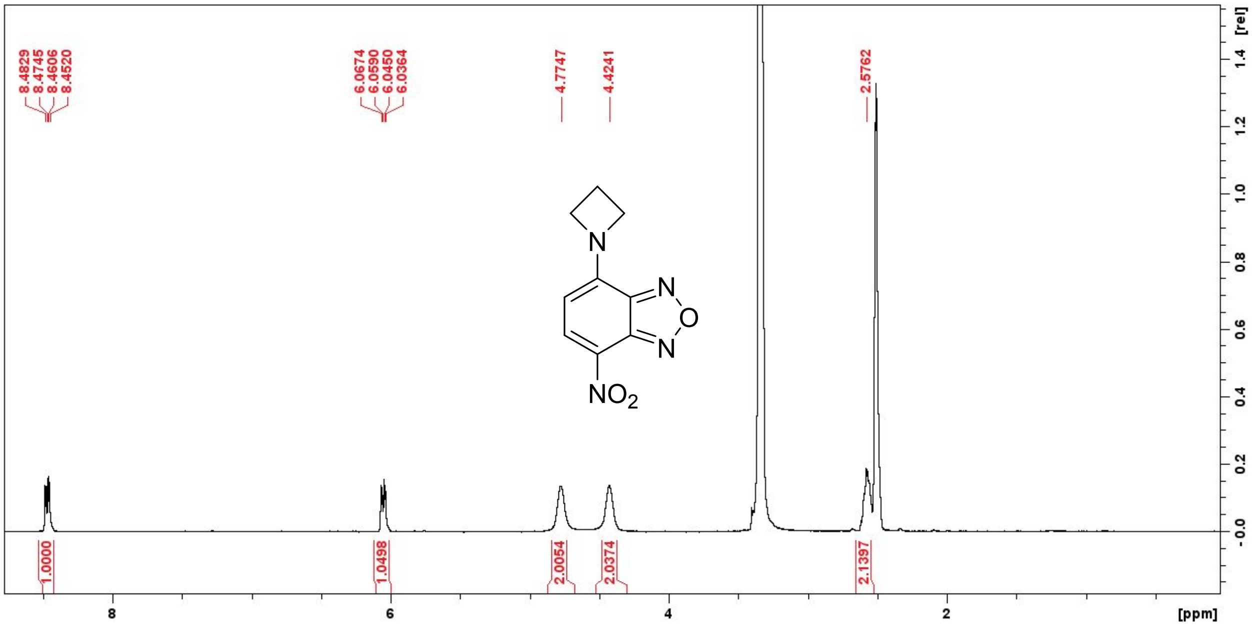

Figure S6. ^{1}H NMR spectrum of **4** in DMSO-d_6.

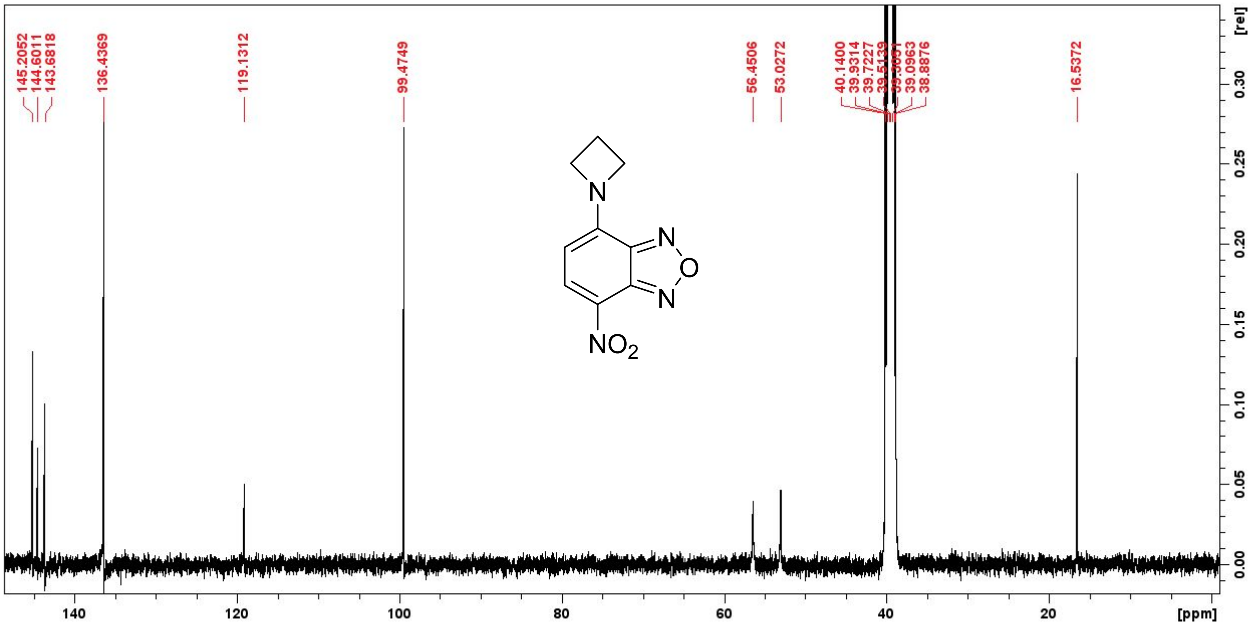

Figure S7. ^{13}C NMR spectrum of **4** in DMSO-d_6.
